# Chemoresistance and Metastasis in Breast Cancer Molecular Mechanisms and Novel Clinical Strategies

**DOI:** 10.3389/fonc.2021.658552

**Published:** 2021-07-01

**Authors:** Jun Cao, Mengdi Zhang, Bin Wang, Long Zhang, Fangfang Zhou, Meiyu Fang

**Affiliations:** ^1^Key Laboratory of Head & Neck Cancer Translational Research of Zhejiang Province, Department of Rare and Head and Neck Oncology, Institute of Cancer Research and Basic Medical Sciences of Chinese Academy of Sciences, Cancer Hospital of University of Chinese Academy of Sciences, Zhejiang Cancer Hospital, Hangzhou, China; ^2^Ministry of Education (MOE) Laboratory of Biosystems Homeostasis & Protection and Innovation Center for Cell Signaling Network, Life Sciences Institute, Zhejiang University, Hangzhou, China; ^3^Institutes of Biology and Medical Science, Soochow University, Suzhou, China

**Keywords:** breast cancer, chemoresistance, metastasis, mechanism, novel strategy

## Abstract

Breast cancer is the most common malignant tumor in females worldwide. Chemotherapy is the standard breast cancer treatment; however, chemoresistance is often seen in patients with metastatic breast cancer. Owing to high heterogeneity, the mechanisms of breast cancer chemoresistance and metastasis have not been fully investigated. The possible molecular mechanisms of chemoresistance in breast cancer include efflux transporters, signaling pathways, non-coding RNAs, and cancer stem cells. However, to overcome this hurdle, the use of novel clinical strategies such as drug carriers, immunotherapy, and autophagy regulation, are being investigated. The goal of this review is to summarize the current data about the molecular mechanisms of breast cancer chemoresistance and the novel clinical strategies; thus, providing a useful clinical tool to explore optimal treatment for breast cancer.

## Introduction

Breast cancer (BRCA) is the most common malignancy and the most frequent cause of cancer-related deaths among women worldwide (1). BRCA is a complex heterogeneous disease classified into three basic types based on the presence or absence of molecular biomarkers for estrogen or progesterone receptors and human epidermal growth factor 2 (ERBB2; formerly HER2). These molecular biomarkers are hormone receptor positive/ERBB2 negative (HR+/ERBB2-; 70% of patients), ERBB2 positive (ERBB2+; 15%-20%), and triple-negative (tumors lacking all 3 standard molecular markers; 15%) (2, 3).

Clinically, the main treatment methods for BRCA include surgery, radiotherapy, chemotherapy, endocrine therapy and targeted therapy (2). Despite that, BRCA is curable in 70%-80% of patients in early stage, non-metastatic disease. The chemoresistance and metastasis in some BRCAs, especially in triple-negative breast cancer (TNBC), are still inevitable and lead to poor prognosis. Chemoresistance is the insensitivity of cancer cells to therapy, which is a key factor resulting in reduced efficacy of anti-BRCA chemotherapy (4). Although various attempts have been made to restore the sensitivity of existing chemotherapeutic drugs and to overcome drug resistance in BRCA, the effects are still unsatisfactory.

This review will summarize the current understanding of chemoresistance mechanisms in BRCA and further discuss the potential of novel clinical strategies to overcome chemoresistance.

## Chemoresistance Mechanisms in BRCA

Chemotherapy is currently the major systemic treatment for BRCA, but unfortunately, patients often develop resistance. The mechanisms of chemoresistance in BRCA urgently need better understanding.

### Efflux Transporters

**Figure 1:**
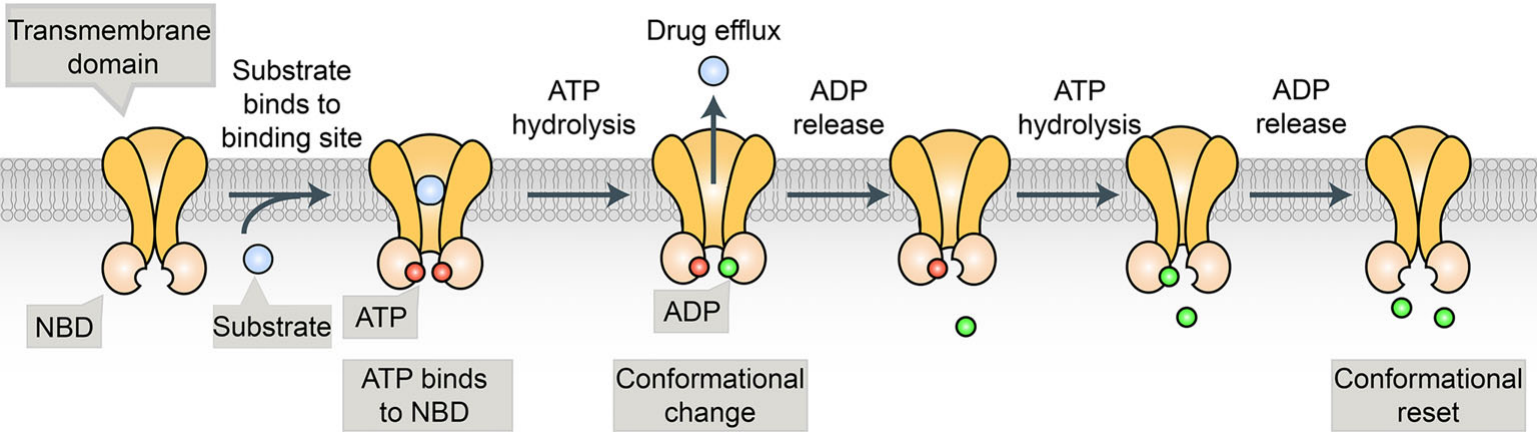
The substrate binds to the binding pocket in TMDs and ATP binds to the two binding sites in the NBDs. This is followed by the hydrolysis of ATP that generates a conformational change, allowing the substrate to be released from the protein. The second molecule of ATP is hydrolyzed, allowing for a conformational reset, where substrate and ATP can bind again so the process can repeat.

Many cancer cells are resistant to a broad spectrum of anticancer drugs through a phenomenon called multidrug resistance (MDR). The major mechanism of MDR is the expression of a class of ATP binding cassette (ABC) transporters. ABC transporters use ATP to pump chemotherapeutic drugs out of cancer cells and decrease intracellular accumulation of anticancer drugs (5) (**Figure 1**). Ample evidence shows that the expression of ABC transporters is strongly implicated in the chemoresistance of numerous solid tumors, including BRCA (5). In the past three decades, at least 15 human ABC transporters have been showed to efflux cancer drugs in some context (5–10) (**Table 1**). In this review, we focus on the subset of ABC transporters that were first reported as multidrug efflux pumps, including ABCB1 [P-glycoprotein/P-gp/MDR1), ABCG2 (BRCA Resistance Protein/BCRP), and ABCC1 (multidrug resistance protein 1(MRP1)] (5, 6).

**Table 1:** ABC transporters and MDR.

Gene	Tissue Localization	Chemotherapeutic Drugs Efflux by Transporter	Clinical significance	Reference
ABCA1	Nervous and hematopoietic system as well as kidney, liver and the blood brain barrier	Cisplatin, doxorubicin	Glioma, lung, testis, liver, colorectal, pancreatic, breast, renal cancer, Tangier disease	(8–10)
ABCA2	Nervous system	Mitoxantrone, estramustine, methotrexate	Alzheimer's disease, melanoma, breast, breast, liver, colon cancer, leukaemia	(8–10)
ABCB1	Small intestine, liver, kidney placenta, blood brain barrier	Anthracyclines, actinomycin D, methotrexate, etoposide, mitomycin C, mitoxantrone, vincristine, vinblastine, taxanes, imatinib, nilotinib, EGFR TKI	Ovarian, breast, colorectal, kidney, adrenocortical cancer, AML	(6, 7, 9, 11, 12)
ABCB4	Liver	Daunorubicin, digoxin, paclitaxel, vinblastine	Liver, lung, pancreatic, renal cancer, melanoma, soft tissue sarcoma	(8, 9)
ABCB5	CD133+ expressing progenitor cells among human epidermal melanocytes	Doxorubicin, 5-fluorouracil, camptothecin, mitoxantrone,	Renal cancer, melanoma	(8, 9, 13)
ABCC1	Lung, testes, peripheral blood monocellular cells	Anthracyclines, etoposide, camptothecins, methotrexate, mitoxantrone, vincristine, vinblastine, irinotecan, TKI as imatinib	Breast, lung, ovarian or prostate cancer, neuroblastoma	(6, 7, 9, 12,14)
ABCC2	canalicular membrane of liver cells, kidney proximal tubule epithelial cells, enterocytes of the small and large intestine	Vinblastine, cisplatin, doxorubicin, methotrexate, paclitaxel	Colorectal, liver, lung, gastric cancer, Dubin-Johnson syndrome	(8, 9, 13)
ABCC3	Liver, intestine, colon, prostate, testes, brain, kidney	Cisplatin, doxorubicin Methotrexate, etoposide, vincristine	Pancreatic, liver, lung, colorectal, stomach, renal, breast cancer	(8, 9)
ABCC4	Widely-expressed	6-mercaptopurine, 6-thioguanine, methotrexate, topotecan	Prostate, renal,liver, lung, breast, ovarian, stomach cancer, neuroblastoma	(8, 9)
ABCC10	Pancreas, liver, placenta, lungs, kidneys, brain, ovaries, spleen, heart	Paclitaxel, docetaxel, vincristine, vinblastine, vinorelbine, cytarabine, gemcitabine	breast, lung, colon, ovarian, and pancreatic cancer	(7, 9)
ABCG1	Pancreas, liver, colon, kidney, brain, lung, lymph nodes, testis	Doxorubicin	Lung, renal, breast, endometrial, prostate, colorectal, cervical, pancreatic cancer, glioma	(9)
ABCG2	Placenta, intestine, liver, colon, breast	Methotrexate, mitoxantrone, topotecan, anthracyclines, irinotecan, methotrexate, paclitaxel, TKI	Liver, testis, prostate, renal, non-small-cell lung cancer, glioma, Alzheimer's disease	(7, 8)

P-gp is the first identified and the most well-investigated protein, which is encoded by a single polypeptide chain with two homologous nucleotide binding domains (NBDs) and two homologous transmembrane domains. A plethora of clinically indispensable chemotherapeutic drugs such as taxol, vincristine, etoposide, and daunorubicin, are susceptible to P-gp-mediated efflux (11, 15–17). Thus, P-gp has been recognized as a promising strategy to overcome MDR and effectively treat cancer (15, 18). In the past 30 years, several P-gp inhibitors or modulators have been investigated in clinical trials in the hope of circumventing MDR, with only limited success (15, 19, 20). Presently, many drug development programs focus on the discovery of new compounds or strategies to bypass the activity of P-gp.

BRCP is the second member of subfamily G within the large human ABC transporter superfamily, which is strongly implicated in the chemoresistance of stem cells in TNBC. As an efflux pump showing a broad substrate specificity localized on the cellular plasma membrane, BCRP excretes a variety of chemotherapeutic agents, such as mitoxantrone, doxorubicin, SN-38, and several TKIs (12, 21). In contrast to the extensive clinical development of P-gp inhibitors, few small-molecule inhibitors specific to BCRP have been tested in clinical trials to date. Zhang et al. (22) found that regorafenib significantly sensitized BCRP-mediated MDR by increasing their intracellular accumulation.

MRP1 is distributed on the membrane of tumor cells. This induces drug resistance by mediating intracellular drug excretion and altering intracellular drug redistribution. Despite the limited sequence identity with P-gp, MRP1 and P-gp have significant substrate overlap. Nevertheless, MRP1 has been shown to transport various neutral and anionic hydrophobic compounds and products of phase II drug metabolism, including many glutathione and glucuronide conjugates (5, 23, 24). In addition, multidrug 88 resistant protein-8 (ABCC11/MRP8) was overexpressed in TNBC and conferred resistance to 5-Fluorouracil and methotrexate (25, 26). Lin et al. (27) reported that histone methyltransferase KDM5c [Lysine(K)-specific demethylase 5C] might downregulate ABCC1 expression by demethylating ABCC1 H3K4me3 in colon cancer.

To date, clinical data about ABC transporter inhibitors in BRCA are still limited. However, the known data support the idea that further research on ABC transporters will be essential in overcoming cancer MDR and in designing strategies against TNBC chemoresistance.

### Signaling Pathway

**Figure 2:**
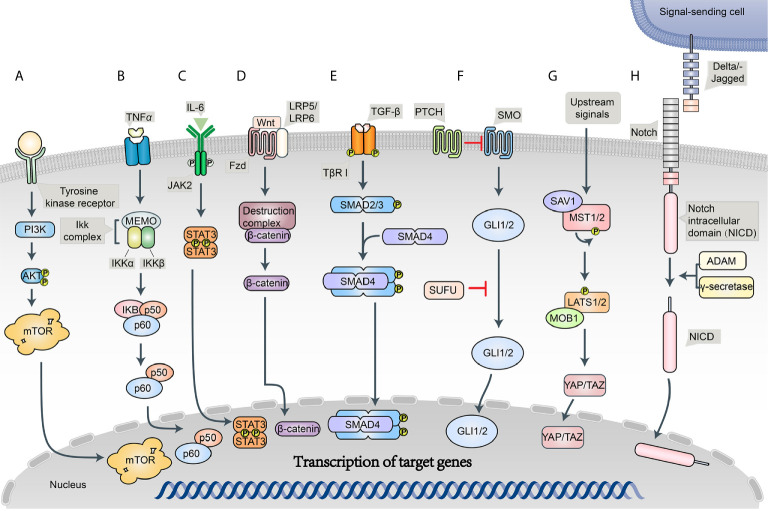
Schematic diagram of the BC signaling pathways. **(A)** PI3K/Akt/mTOR signaling pathway; **(B)** NF-κB signaling pathway; **(C)** JAK/STAT signaling pathway; **(D)** Wnt/Frizzled/β-catenin signaling pathway; **(E)** Notch signaling pathway; **(F)** Hedgehog (Hh) signaling pathway; **(G)** Hippo signaling pathway; **(H)** TGF-β signaling pathway.

An intricate network of signaling pathways governs the survival, growth, and invasion of BRCA. PTEN/PI3K/AKT/mTOR, NF-κB, and JAK/STAT are implicated in chemotherapy resistance to BRCAs (11, 16) (**Figure 2**).

PI3K-AKT-mTOR (PAM) pathway is one of the critical mechanisms of cells control survival, growth, proliferation, and motility. Phosphoinositide 3-kinase (PI3K) is a heterodimeric molecule from a larger family of lipid kinases that phosphorylate 3-hydroxyl group of phosphoinositides, which can activate AKT kinase by regulating phosphatidylinositol-3,4,5-triphosphate (PIP3) (28). The activation of AKT shows an important indirect effect on the phosphorylation of mammalian target of rapamycin (mTOR), which will in turn enhance protein synthesis and cell growth, giving malignant cells a significant advantage (16, 28). PAM activity is negatively regulated by tumor suppressor phosphatase and tensin homolog (PTEN). Because of PTEN loss and high AKT expression, PAM pathway is often associated with aggressive tumors, poor prognosis, and chemoresistance in BRCA (16, 28, 29). Several drugs targeting PI3K/ATK/mTOR are currently in clinical trials, in combination with endocrine therapy and anti-HER2 therapy (28, 30).

The activation of nuclear factor-kappa B (NF-κB), a proinflammatory transcription factor, is a commonly observed phenomenon in BRCA. NF-κB family consists of five members, namely, RelA (p65), c-Rel, RelB, NFκB1 (p50), and NFκB2 (p52), which form homo- and heterodimers to activate the transcription of target genes regulating host inflammatory and immune responses as well as cellular growth and survival (16, 31). Moreover, it is well established that NF-κB signaling pathway is a crucial regulator of TNBC and is associated with chemoresistance and metastasis in BRCA (16, 32, 33). Ekambaram et al. (34) showed that NF-κB activation promotes the aggressiveness of BRCA. Kastrati at al. (35) reported that NF-κB pathway promotes tamoxifen tolerance and disease metastasis in estrogen receptor-positive BRCAs.

JAK/STAT pathway was originally discovered as an evolutionarily conserved cellular mechanism mediating the actions of cytokines, interferons, and growth factors and as well control their gene expression (36). The activation of JAK/STAT pathway in tumor cells is known to contribute to tumor growth and progression. Both STAT3 and STAT5 have been shown to promote BRCA growth and progression, and JAK/STAT pathway has been found to be a potential therapeutic target in BRCA patients (37, 38).

Additionally, some signaling pathways, which play essential roles in cancer stem cell self-renewal, represent a promising approach to control chemoresistance and metastasis of BRCAs.

Wnt/β-catenin pathway is an important regulator of normal breast development and abnormal tumorigenesis. Wnt signaling proteins interact with the frizzled family of cell-surface receptors and activate the proteins of the disheveled family, which in turn results in the inhibition of proteolytic degradation of β-catenin. Subsequently, stabilized β-catenin is translocated into the nucleus, leading to the transcription of target genes such as C-Myc and Cyclin D1, which are involved in determining cell migration, cytoskeletal activity, cell polarity, and cellular differentiation (39, 40). Recently, the overexpression of Wnt pathway has been observed in breast, lung, and hematopoietic malignancies and contributes to tumor recurrence (41). Multiple Wnt/β-catenin targeted inhibitors were designed in the wake of these studies (42). Hence, the inhibition of Wnt signaling pathway has been proposed as a potential therapeutic strategy to target BRCA.

Notch signaling pathway plays an essential role in normal stem cell maintenance and differentiation, a dysfunction which has been linked to the development of BRCA and is believed to be upregulated in a variety of cancers (43). Canonical notch signaling pathway has four cell surface receptors (Notch 1–4) and five transmembrane ligands (Delta-like 1,3,4 and JAGGED-1,2). These notch cell surface receptors can be activated by membrane-tethered ligands on neighboring cells. The activation of cell surface receptors induces successive cleavages by ADAM proteases and γ-secretase, resulting in the release of intracellular domain (NICD) of the receptor, which is in turn translocated to the nucleus and regulates context-specific patterns of cancer-related gene expression (44, 45). Therapeutic resistance in BRCA is also believed to be associated with the notch signaling pathway. Previous studies have confirmed that notch signaling is crucial in chemoresistance and have demonstrated the ability of notch inhibitors to sensitize cells, including BRCA and cytotoxic agents (46, 47). Further investigation on notch inhibitors has been an area of strong interest in cancer research.

Hedgehog (Hh) signaling pathway plays a crucial role in embryonic development, tissue regeneration, and stem cell renewal. Hh pathway consists of three secreted ligands (Sonic-SHH, Indian 159 IHH, and Desert-DHH), which bind transmembrane receptor/co-receptors Patched (PTCH) and Smoothened (SMO). Three glioma-associated oncogene transcription factors (GLI1–3) are the main effectors that regulate the expression of many target genes, such as ABCG2 and VEGF (48, 49). In mouse models of TNBC, hedgehog ligand produced by neoplastic cells reprograms cancer-associated fibroblasts (CAFs) to provide a supportive niche for the acquisition of chemoresistance (50). Moreover, the combination of hedgehog pathway inhibitors and itraconazole was observed to improve the prognosis of BRCA (51).

Hippo signaling pathway is important in regulating tissue homeostasis, organ size, and tumorigenesis. Hippo signaling is modulated *via* two pairs of kinases, Mst1/2 and Lats1/2. Upon the phosphorylation of downstream Yes-associated protein 1 (YAP1) or Lats1/2-induced TAZ, transcription is inactivated and leads to cellular degradation, whereas dephosphorylation leads to YAP/TAZ nuclear translocation and subsequent activation of transcription (52). Dysregulation of hippo pathway leading to the overexpression of YAP1 or TAZ has been seen in many types of cancer (53, 54). Furthermore, some studies have provided evidence that YAP acts as a promoter of focal adhesion and tumor invasiveness by regulating FAK phosphorylation in BRCA (55).

Transforming growth factor-β (TGF-β) is a member of a large cytokine superfamily that consists of over 30 related growth factors, including three TGF-β isoforms (TGF-β1–3) (16). TGF-β exerts its cellular effects *via* TGF-β type I and type II cell surface receptors (TβRI/II). TGF-β initially engages in TβRII, which subsequently drives the recruitment of TβRI and the formation of a heterotetrameric complex. The activation of TβRI, causes the recruitment and phosphorylation of the main effectors of this pathway, Smad2 and Smad3, which interact with Smad4 to form a heteromeric complex that is transported into the nucleus to regulate a series of genes, such as ANGPTL4, CTGF, IL11, S100A4, and PTHrP, and further facilitates cancer cell migration and invasion (56, 57). In oncology, TGF-β appears to have a dual function, where it represses early tumor growth but promotes metastasis in advanced stages. However, the mechanism by which TGF‐β switches its role from a tumor inhibitor to a cancer promoter remains unclear (58).

In conclusion, the crucial role of the developmental pathways in BRCA initiation, progression, metastasis, and chemoresistance is undeniable. Because of the considerable crosstalk and collaboration existing in this signaling network, successful targeted medicines still need further research.

### Non-Coding RNAs (ncRNAs)

Non-coding RNAs (ncRNAs) are the regulators of intracellular and intercellular signaling in BRCA (59). Owing to the development of next-generation sequencing technologies, ncRNAs, including long non-coding RNAs (lncRNAs), microRNAs (miRNAs), and circular RNAs (circRNAs), play essential roles in chemoresistance in BRCA.

miRNAs are the major class of endogenous, small ncRNA molecules of 18–25 nucleotides in length. Recent studies have shown that dysregulated miRNAs often cause the development of metastasis and chemoresistance in BRCA. Li et al. (60) demonstrated that the overexpression of miR-770 inhibited doxorubicin resistance and metastasis *in vivo*. Further experiments confirmed that miR-770 regulates chemoresistance and metastasis by targeting STMN1 in BRCA. Rodriguez et al. (61) found that loss of miR-424(322)/503 promotes chemoresistance in BRCA *via* the overexpression of two of its targets: BCL-2 and insulin-like growth factor-1 receptor (IGF1R). In addition, a novel miR-20a/MAPK1/c-Myc feedback loop was reported to significantly regulate BRCA growth and chemoresistance (62). Based on these findings, some researchers proposed that the combined use of miRNAs and chemotherapeutic agents might be a promising therapeutic strategy to increase long-term drug responses in BRCAs, especially for chemo-resistant patients (62–64).

lncRNAs are greater than 200 nucleotides and sometimes are 100 kb long. Recent research verified the involvement of lncRNA-small nucleolar RNA host gene 14 (SNHG14) in the mediation of trastuzumab responses *via* tumor cell extracellular exosomes. The expression level of serum exosomal lncR-SNHG14 was upregulated in patients who showed resistance to trastuzumab and the knockdown of lncR-SNHG14 potently promoted trastuzumab-induced cytotoxicity (65). In another study, Dong et al. (66) confirmed that lncRNA AGAP2-AS1 could promote BRCA growth and trastuzumab resistance by activating NF-κB signaling pathway and upregulating MyD88 expression. High expression of lncRNA AGAP2-AS1 was associated with poor clinical response to trastuzumab therapy in BRCA patients. Furthermore, Yao et al. (67) reported that novel lncRNA NONHSAT101069 was significantly overexpressed in BC specimens and promoted epirubicin resistance. lncRNA cancer susceptibility candidate 2 (CASC2) and lncRNA ferritin heavy chain 1 pseudogene 3 (FTH1P3) were found to activate paclitaxel resistance in BRCA through the regulation of miRNA (68, 69).

circRNAs are a group of ncRNAs formed by covalently closed loops through back-splicing. The latest study reported that circRNAs are key regulators in the development and progression of human cancers (70). In vitro loss-of-function experiments showed that circ-ABCB10 knockdown suppressed the proliferation and increased the apoptosis of BRCA cells by sponging miR-1271 (71). Circ 222 ANKS1B was significantly overexpressed in TNBC tissues compared to normal BRCA tissues, which promoted BRCA invasion and metastasis by inducing epithelial-to-mesenchymal transition (EMT) (72). Du et al. (73) reported that circ-Dnmt1-mediated autophagy is essential in enhancing BRCA progression. High expression of circular RNA circ-Dnmt1 could bind to and regulate oncogenic proteins in BRCA cells.

### Cancer Stem Cells (CSCs)

**Figure 3:**
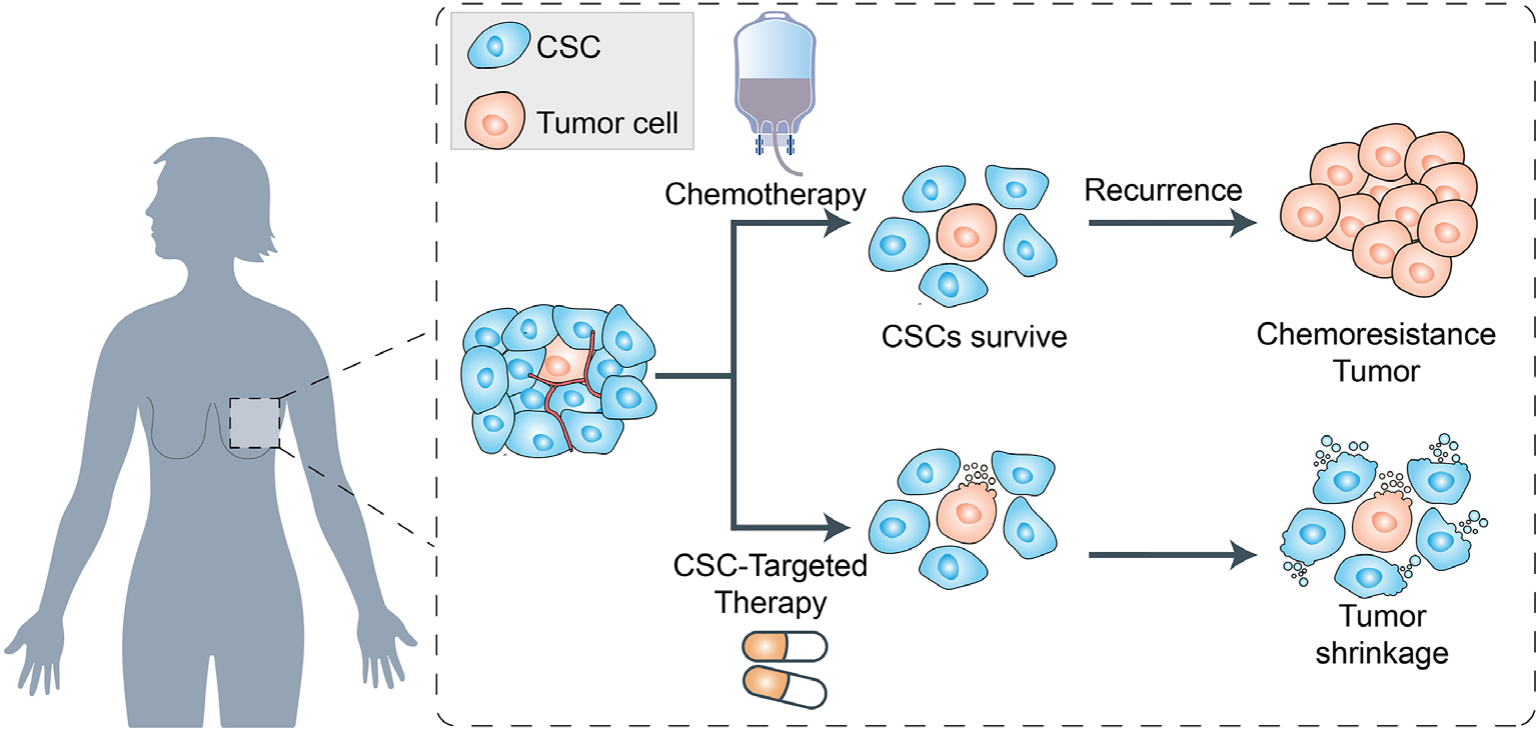
Schematic representation of cancer stem cells (CSCs) and their role in chemoresistance. The cancer stem cells (CSCs), not only enable tumor formation and progression but also mediate tumor metastasis and therapeutic resistance. On the one hand, the CSCs survived from chemotherapy will gain the chemoresistance and enhance the key processes involved in cancer progression. On the other hand, the cancer therapeutics targeted to CSCs biomarkers which can modulate EMT and CSC properties, can lead to the tumor shrinkage in clinical therapeutics.

There is substantial evidence that BRCAs are driven by a population of cells that display stem cell properties. This small subset of tumorigenic cells termed cancer stem cells (CSCs), not only enable tumor formation and progression but also mediate tumor metastasis and therapeutic resistance (13, 74). Previous studies have shown that BRCA stem cells (BCSCs) overexpress various ABC transporters such as P-gp, ABCG2, ABCC1, and ABCB5 (11, 14). Studies have shown that these transporters can help BCSCs to pump out chemotherapeutic agents and enhance the key processes involved in cancer progression (75, 76) (**Figure 3**).

In addition, a series of BCSC surface biomarkers such as CD10, CD24, CD44, CD133, GPR77, ALDH1, EpCAM, and ABCG2 have been confirmed, and their overexpression is an important cause of BCSC chemoresistance (11). Su et al. (77) demonstrated that two cell-surface molecules, CD10 and GPR77, can promote tumor formation and chemoresistance by providing a survival niche for BCSCs. Moreover, Li et al. (78) found that both high CD44/CD24 ratio and ALDH1+ were conserved during metastasis. These results confirmed the potential of these BCSC biomarkers in monitoring tumor progression, metastasis, and even in cancer therapeutics.

These therapeutic targets, which can modulate EMT and CSC properties, may be utilized in clinical therapeutics.

## Novel Clinical Strategies

### Novel Drug Delivery System

Presently, endocrine therapy is the main therapy for hormone-responsive or receptor-positive BRCA. However, poor solubility and bioavailability, lack of intracellular transport within cancer cells, and development of chemoresistance are the problems associated with conventional therapies for BRCA, especially TNBC (11, 29). Hence, novel drug delivery systems are being explored to fight this lethal disease.

Nanocarriers, including nanoparticles, nanoscale, and liposomes, have been shown to have the advantages of targeted drug release, prolonged blood circulation, enhanced synergies, and superior biocompatibility (79). Zhang et al. (80) developed a core-shell nanocarrier coated with cationic albumin to simultaneously deliver miRNA-34a and docetaxel (DTX) into BRCA cells. The co-delivery nanocarriers prolonged the blood circulation of DTX, enhanced tumor accumulation of cargo, and significantly inhibited tumor growth and metastasis both *in vivo* and *in vitro*. Bose et al. (81) investigated a tumor cell-derived extracellular vesicle-based nanoplatform for multimodal miRNA delivery and phototherapy treatments, which attenuated doxorubicin (DOX) resistance in BRCA cells with a 3-fold higher cell killing efficiency than in cells treated with DOX alone. Gong et al. (82) developed a strategy to produce nanoscale target-specific Exo to co-deliver cholesterol-modified miRNA and chemotherapeutic drugs to TNBC cells, which showed improved anticancer effects, without adverse effects. Furthermore, some researchers explored the natural ability of macrophages to target cancer cells through extracellular vesicles (EVs) as drug delivery vehicles. Haney et al. (83) reported that drug loaded EVs can target TNBC *in vivo* and abolish tumor growth. In another study, Tang et al. (84) assessed the feasibility of liposomal drug delivery system combining bevacizumab and chemotherapy for the treatment of HER2/MDR double-positive BRCA cells. In HER2 positive and multidrug resistant BRCA cell mouse model, tumor size decreased steadily within 60 days.

Nanomedicine helps in in bringing major advances in the chemoresistance and metastasis in BRCAs. Looking into the future, the use of nanomedicine, combining anticancer targeted therapy and multifunctional nanocarriers that contain therapeutic and imaging agents, might become promising cancer treatments to achieve the goal of personalized medicine based on the needs of an individual patient or cell subpopulation and overcome the chemoresistance.

### Novel Anticancer Drugs

#### Immune Checkpoint Inhibitors (ICIs)

Immunotherapy is a promising treatment for multiple solid tumors using the patient’s own immune system directly to target and eradicate neoplastic cells. Early data have revealed the clinical activity of immune checkpoint inhibitors (ICIs), which mainly target programmed cell death protein 1(PD-1) and cytotoxic T lymphocyte-associated protein 4 (CTLA-4) in small number of metastatic BRCA patients (85, 86).

PD-1 is an inhibitory immune checkpoint inhibitor that limits T-cell effector function within the tissues and is expressed on the surfaces of immune effector cells. Adams et al. (87) assessed the safety and antitumor activity of PD-1 inhibitor pembrolizumab in patients with PD-L1-positive advanced TNBC. The median duration of response was 10.4 months. The median PFS was 2.1 months (95% CI 2.0-2.2), and the median overall survival was 18.0 months (95% CI 12.9-23.0). Emens et al. (88) evaluated the clinical activity and safety associated with the use of single-agent atezolizumab (anti programmed cell death ligand 1 (PD-L1)) in patients with metastatic TNBC. The result showed that median PFS was 1.4 months (95% CI, 1.3-1.6 months) and median OS was 17.6 months (95% CI, 10.2 months and above). Based on these results, PD-1 antagonists have a manageable safety profile and show durable antitumor activity as first-line therapy for patients with PD-L1-positive BRCA.

CTLA-4 is a T-cell inhibitory receptor that is expressed on activated CD8^+^ T cells and CD4^+^regulatory T cells that express CD25 and FOXP3. Therefore, CTLA-4 inhibitors induce anti-tumor immunity by blocking FOXP3+ Treg cells, resulting in enhanced inhibition of tumor cells (89). Currently, ipilimumab and tremelimumab (two promising anti-CTLA-4 antibodies) have been used in clinical trials related to TNBC (90). Nanoparticle-based mRNA vaccine and CTLA-4 inhibitor for TNBC have also been demonstrated as a potential strategy (91). Moreover, Pai et al. (92) developed a dual variable domain immunoglobulin of anti-CTLA4 antibody that can help deplete tumor-infiltration, but not tissue-resident Tregs, preserving antitumor effects while minimizing toxicity.

#### Cyclin-Dependent Kinase 4/6 (CDK4/6) Inhibitors

The cyclin D/cyclin-dependent kinases 4 and 6 (CDK4/6)–retinoblastoma protein pathway plays a key role in the proliferation of both normal breast epithelium and BRCA cells (93). Abemaciclib is the most potent inhibitor of CDK4 and CDK6 and shows promising clinical activity in metastatic BRCA. In a phase II study, Dickler et al. (94) evaluated the single-agent activity and safety of abemaciclib in women with HR^+^/HER2^−^ metastatic BRCA. The result showed the objective response rate was 19.7%, clinical benefit rate of 42.4%, median PFS of 6.0 months, and median OS of 17.7 months, which confirmed the striking activity of abemaciclib as a single agent. In a neoadjuvant phase II study, Palbociclib, another CDK4/6 Inhibitor, was found to overcome intrinsic endocrine resistance in primary BRCA (95). Moreover, the combination of Palbociclib and Letrozole resulted in significantly longer PFS than monotherapy among patients with advanced BRCA (96).

#### Combination Therapy

Compared to single-agent therapy, combination treatment regimens may provide a more efficacious solution to BRCA resistance. The combination of abemaciclib, fulvestrant, and trastuzumab has been found to improve PFS and prognosis in patients with advanced BRCA (97). Teo et al. (98) reported that combined PI3Kα and CDK4/6 inhibition is synergistically effective against multiple TNBC models by increasing apoptosis, cell-cycle arrest, and tumor immunogenicity and generating immunogenic cell death. In a Phase I trial, Clark et al. (99) enrolled cohorts of patients to sequentially ingest oral doses of Palbociclib intermittently between days 1 and 19 of a 28-day cycle alternating with weekly paclitaxel. The result showed that the combination of paclitaxel and palbociclib is feasible and safe, without evidence of additive toxicity in patients with advanced BRCA. In addition, atezolizumab and nab-paclitaxel have been confirmed to prolong PFS among patients with metastatic TNBC (100). The combination of tucatinib, trastuzumab, and capecitabine has also been reported to improve PFS and OS outcomes in HER2^-^ positive metastatic BRCA patients (101).

Overall, it is obvious that immunotherapy is emerging as a novel promising option for TNBC. However, further investigations are required to completely determine the safety and effectiveness of these immunotherapies and eventually define the most effective combination regimens for the treatment of TNBC.

### Autophagy Regulation

**Figure 4:**
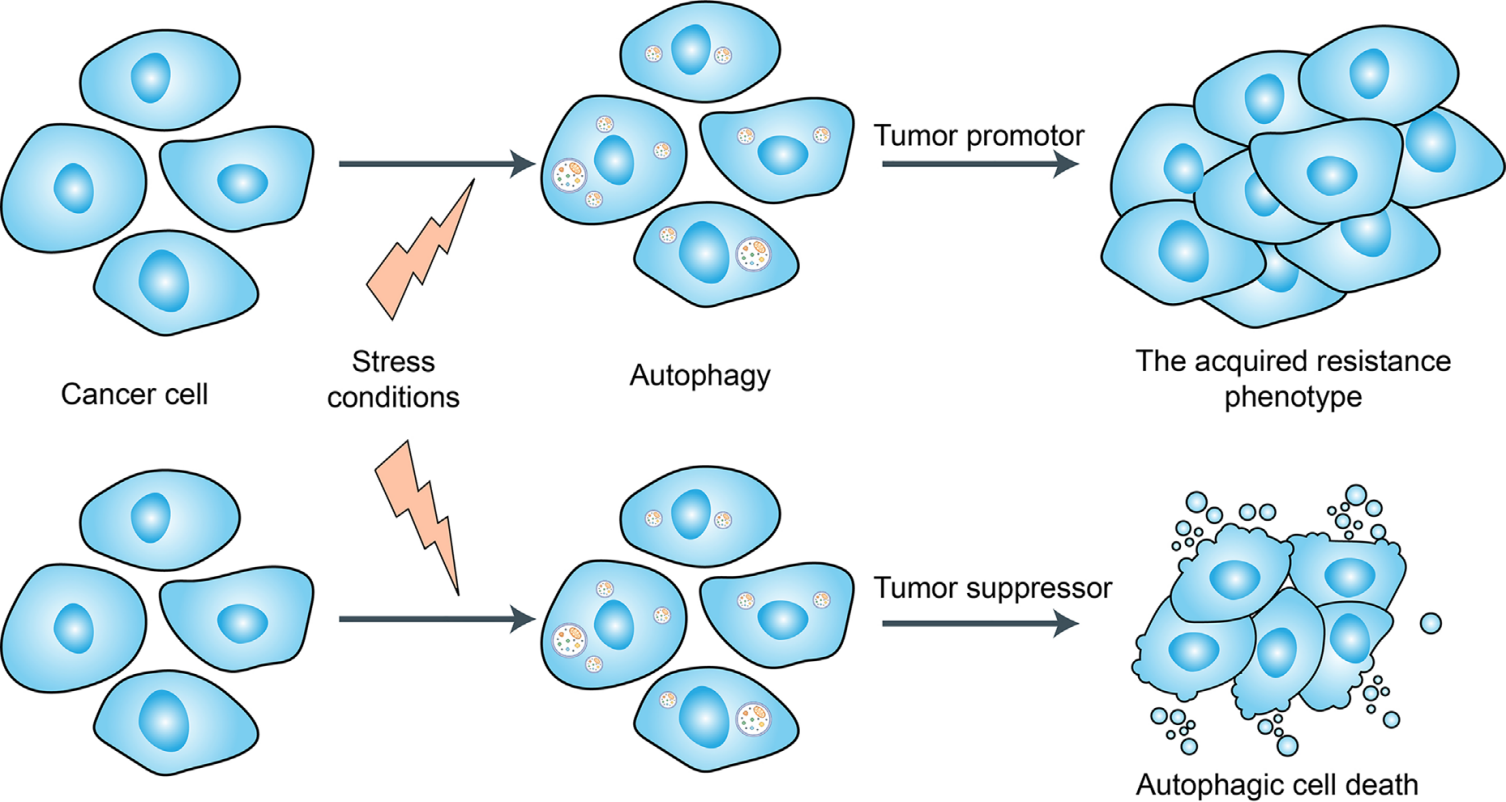
Dual role of autophagy for therapeutic purposes in cancer. Autophagy is induced in tumors by many different stress conditions including some cancer therapeutic approaches, which function as a death executioner to induce autophagic cell death. But autophagy also provides resistance to cancer cells against chemo-/radio-therapies and cell death.

Autophagy is a tightly regulated catabolic process that facilitates nutrient recycling from damaged organelles and other cellular components through lysosomal degradation and provides energy and macromolecular precursors (102) (**Figure 4**). Substantial evidence has indicated that autophagy plays a dual role in the regulation of chemoresistance in cancer patients by either promoting drug resistance or increasing drug sensitivity (103, 104). Hydroxychloroquine (HCQ) is the only clinically approved autophagy inhibitor that increases tumor cell death alone or in combination with targeted agents or cytotoxic chemotherapy (103, 104). In a recent study, Cook et al. (105) demonstrated that HCQ can increase antiestrogen responsiveness in ER+ BRCA through the inhibition of autophagy and the combination of HCQ and tamoxifen showed a positive outcome for ongoing neoadjuvant clinical trials. Furthermore, with the development of nanotechnology, nanomaterials can modulate autophagy and have been exploited as therapeutic agents against cancer (106). Although, autophagy inhibition has been suggested as a promising approach for chemoresistance in BRCAs, due to the lack of organ-specificity, the utilization of autophagy-related kinase inhibitors/activators may also lead to uncontrolled side effects. Whether these agents of autophagy regulation will eventually be used in the clinic still requires further study.

### BCSC-Directed Therapy

Increasing evidence shows the existence of tumor initiating or cancer stem cells within tumors that are responsible for drug resistance, cancer recurrence, and cancer metastasis. Currently, novel anti-BCSCs drugs, targeting the Wnt/Frizzled/β-catenin, notch and hedgehog pathways have reached clinical trials for BRCA patients (14). The most clinically evolved approach is the inhibition of notch signaling using γ-secretase inhibitors (GSIs). At present, researchers have shown that GSIs can inhibit BRCA growth in a process that is coupled with IL6 induction and thus might serve as a novel therapeutic strategy for treating patients with BRCAs (107, 108). Other inhibitors of Notch signaling, such as CB-103, are also currently in phase I/II clinical trials for advanced or metastatic BRCA (14). In addition, the Wnt/Frizzled/β-catenin pathway is overactivated in TNBC and several other cancers. Wnt inhibitors work to eradicate the tumor resistant stem cell and thus may overcome resistance to conventional therapy (39). Ahmed et al. (109) reported that an anti-leprotic drug clofazimine is effective against TNBC by specifically inhibiting canonical Wnt signaling. Inhibitors of hedgehog pathway have also been explored *in vitro* and *in vivo*, but their efficacy in BRCA has been disappointing (49). In summary, accumulating evidence has shown the potential efficacy of targeting BCSCs in reversing drug resistance *in vitro* and *in vivo*. However, the majority of studies are still in the early stages. Thus, continuing effort in establishing clinically relevant biomarkers of BCSC is urgently needed for translating the knowledge from laboratory to clinical practice.

## Conclusion

With the rapid development of molecular biology, great progress has been achieved in breast cancer treatment; however, some groups of BRCA, such as TNBC, display significant problems of chemoresistance and metastasis. Owing to the complexity of BRCAs, completely understanding the molecular mechanisms of BRCA remains a significant challenge, however, is vital for the identification of new treatment targets. Currently, novel treatment regimens have been proven as a more efficient solution to BRCA resistance than conventional therapy. The exploration of novel delivery systems has provided a potential approach to improve the effectiveness of anti-cancer agents in cancers with chemoresistance. Moreover, the progress of immunotherapy offers a promising alternative for drug-resistant tumors, and further research is needed to explain the complex mechanisms of tumors. Although the regulation of autophagy and cancer stem cells has not been widely used clinically it is hopeful to improve the prognosis of BRCA with chemoresistance and metastasis. In conclusion, future clinical studies on BRCA are needed, with a focus on molecular mechanisms. Novel clinical strategies are expected to improve the survival of BRCA patients.

## Author Contributions

CJ and ZM conceived and drafted the manuscript. ZL, FY, and FZ discussed the concepts of the manuscript. ZM drew the figures. LZ and FZ approved the version to be submitted. All authors contributed to the article and approved the submitted version.

## Funding

This work was supported by a special program from the Ministry of Science and Technology of China (2016YFA0502500 to LZ), the Chinese National Natural Science Funds (81702653 to JC; 31925013, U20A20393 and 91753139 to LZ; 31871405 and 82041009 to FZ), Jiangsu National Science Foundation (BK20180043 and 19KJA550003 to FZ), the Zhejiang Natural Science Fund (LD19C070001to LZ), Medical and Health Science and Technology Plan (2018253753 to JC), and A project Funded by the Priority Academic Program Development of Jiangsu Higher Education Institutions. This work was supported by a special program from the Ministry of Science and Technology of China (2016YFA0502500 to LZ), the Chinese National Natural Science Funds (81702653 to JC; 31925013, U20A20393 and 91753139 to LZ; 31871405 and 82041009 to FZ), Jiangsu National Science Foundation (BK20180043 and 19KJA550003 to FZ), the Zhejiang Natural Science Fund (LD19C070001to LZ), Medical and Health Science and Technology Plan (2018253753 to JC), and A project Funded by the Priority Academic Program Development of Jiangsu Higher Education Institutions.

## Conflict of Interest

The authors declare that the research was conducted in the absence of any commercial or financial relationships that could be construed as a potential conflict of interest.
