# Corrigendum: Chemoresistance and Metastasis in Breast Cancer Molecular Mechanisms and Novel Clinical Strategies

**DOI:** 10.3389/fonc.2021.745052

**Published:** 2021-08-17

**Authors:** Jun Cao, Mengdi Zhang, Bin Wang, Long Zhang, Fangfang Zhou, Meiyu Fang

**Affiliations:** ^1^Key Laboratory of Head & Neck Cancer Translational Research of Zhejiang Province, Department of Rare and Head and Neck Oncology, Institute of Cancer Research and Basic Medical Sciences of Chinese Academy of Sciences, Cancer Hospital of University of Chinese Academy of Sciences, Zhejiang Cancer Hospital, Hangzhou, China; ^2^Ministry of Education (MOE) Laboratory of Biosystems Homeostasis & Protection and Innovation Center for Cell Signaling Network, Life Sciences Institute, Zhejiang University, Hangzhou, China; ^3^Institutes of Biology and Medical Science, Soochow University, Suzhou, China

**Keywords:** breast cancer, chemoresistance, metastasis, mechanism, novel strategy

In the published article, there was an error in affiliation 1. Instead of “Department of Rare and Head and Neck Oncology, Institute of Cancer Research and Basic Medical Sciences of Chinese Academy of Sciences, Cancer Hospital of University of Chinese Academy of Sciences, Zhejiang Cancer Hospital, Hangzhou, China.”, it should be “Key Laboratory of Head & Neck Cancer Translational Research of Zhejiang Province, Department of Rare and Head and Neck Oncology, Institute of Cancer Research and Basic Medical Sciences of Chinese Academy of Sciences, Cancer Hospital of University of Chinese Academy of Sciences, Zhejiang Cancer Hospital, Hangzhou, China.”.

In the published article, there was an error in author order. Instead of “Jun Cao, Mengdi Zhang, Bin Wang, Long Zhang, Meiyu Fang and Fangfang Zhou”, it should be “Jun Cao, Mengdi Zhang, Bin Wang, Long Zhang, Fangfang Zhou and Meiyu Fang”.

In the published article, there was an error in corresponding author order. Instead of “Long Zhang L_Zhang@zju.edu.cn; Fangfang Zhou zhoufangfang@suda.edu.cn; Meiyu Fang fangmy@zjcc.org.cn”, it should be “Meiyu Fang fangmy@zjcc.org.cn; Long Zhang L_Zhang@zju.edu.cn; Fangfang Zhou zhoufangfang@suda.edu.cn”.

The authors apologize for these errors and state that this does not change the scientific conclusions of the article in any way. The original article has been updated.

## Publisher’s Note

All claims expressed in this article are solely those of the authors and do not necessarily represent those of their affiliated organizations, or those of the publisher, the editors and the reviewers. Any product that may be evaluated in this article, or claim that may be made by its manufacturer, is not guaranteed or endorsed by the publisher.

